# Protocol for a non-inferiority randomized controlled trial of spontaneous breathing trial in children with and without pressure support

**DOI:** 10.62675/2965-2774.20250187

**Published:** 2025-11-27

**Authors:** José Colleti, Orlei Ribeiro de Araujo, Karina Tavares Weber, Gabriela Maria Virgílio Dias Santos, Dafne Cardoso Bourguignon da Silva, Leila Costa Volpon, Ana Paula de Carvalho Panzeri Carlotti

**Affiliations:** 1 Universidade de São Paulo Faculdade de Medicina de Ribeirão Preto Department of Pediatrics, Hospital das Clínicas Ribeirão Preto SP Brazil Department of Pediatrics, Hospital das Clínicas, Faculdade de Medicina de Ribeirão Preto, Universidade de São Paulo - Ribeirão Preto (SP), Brazil.; 2 Universidade Federal de São Paulo Instituto de Oncologia Pediátrica Grupo de Apoio ao Adolescente e à Criança com Câncer São Paulo SP Brazil Grupo de Apoio ao Adolescente e à Criança com Câncer, Instituto de Oncologia Pediátrica, Universidade Federal de São Paulo - São Paulo (SP), Brazil.

**Keywords:** Respiration, artificial, Spontaneous breathing trials, Palliative care, Critical illness, Continuous positive airway pressure, Child, Intensive care units, pediatric

## Abstract

**Objective:**

To investigate whether continuous positive airway pressure during spontaneous breathing trial is non-inferior to pressure support by comparing both techniques in mechanically ventilated children.

**Methods:**

This is a multicenter, open-label, non-inferiority randomized controlled trial. The primary outcome is successful liberation from invasive mechanical ventilation for at least 48 hours post-extubation. Secondary outcomes include the need for post-extubation respiratory support and the length of stay in the pediatric intensive care unit. The sample size is estimated to be 170 participants. Non-inferiority will be assessed using the Farrington-Manning test. The trial registration number is NCT06593288 (clinicaltrials.gov). Infants older than 36 weeks corrected gestational age and < 18 years old admitted to the pediatric intensive care unit requiring invasive mechanical ventilation for at least 24 hours and ready to wean will be included. Patients with chronic pulmonary conditions, congenital heart disease, upper airway abnormalities, morbid obesity, and in palliative care will be excluded. Patients who have passed the extubation readiness test will be randomized to receive either continuous positive airway pressure or pressure support during a spontaneous breathing trial.

**Results:**

the results of the study should be ready and published within a year.

**Conclusion:**

The transition from mechanical ventilation to spontaneous breathing is a pivotal moment in the care of critically ill children. Yet, limited high-quality evidence informs the optimal approach to spontaneous breathing trials. Our protocol outlines a rigorously designed non-inferiority randomized controlled trial comparing spontaneous breathing trials conducted with and without pressure support.

## INTRODUCTION

While life-saving, invasive mechanical ventilation (MV) is associated with both short- and long-term complications.^(1)^ Once the underlying condition improves, timely weaning from ventilatory support is essential. Weaning is complex, often guided by unit protocols and physician experience rather than standardized techniques. Successful extubation is a key objective, and determining extubation readiness has become a significant focus of clinical research.^(2–4)2^

Although spontaneous breathing trials (SBTs) and extubation readiness tests (ERTs) are frequently used interchangeably, they differ in scope. Spontaneous breathing trials assess the ability to breathe spontaneously with minimal support. At the same time, ERT also evaluates additional factors such as sedation levels, neurological status, and the risk of upper airway obstruction.^(3,5)^ Spontaneous breathing trials have been associated with reduced MV duration, fewer complications, and lower healthcare costs in adults.^(6–10)^ In pediatric populations, daily SBTs have been shown to reduce the duration of MV by up to 30%.^(11)^

A survey of pediatric intensivists revealed that 86% use SBTs before extubation, with 50% following a protocolized approach.^(3)^ Despite its widespread use, the optimal technique and duration of SBT in children remain unclear. Standard methods for pediatric SBT include T-piece, continuous positive airway pressure (CPAP), or CPAP with low-level pressure support (PS).^(5,12)^

The use of PS during SBT is widely debated. Some argue that PS mitigates the resistance imposed by the endotracheal tube, while others claim this is a theoretical benefit without clinical backing. Studies suggest that PS may underestimate the post-extubation work of breathing, potentially leading to a higher rate of false-positive extubation readiness. Despite these concerns, PS remains the preferred method for many pediatric intensivists, supported by adult studies showing its efficacy in predicting extubation success.^(13,14)^

However, robust pediatric data comparing PS with other SBT methods is limited, and recent pediatric ventilator liberation guidelines have only conditionally recommended either PS or CPAP for SBT, citing low-certainty evidence.^(4)^ This trial will investigate whether CPAP during SBT is non-inferior to PS by comparing both techniques in mechanically ventilated children.

### Study design

This multicenter, open-label, non-inferiority randomized controlled trial will compare SBTs with PS and CPAP in mechanically ventilated children. The study "Protocol for a non-inferiority randomized controlled trial of sbt in children with and without pressure support" was approved by the Institutional Review Board of *Hospital das Clínicas* of *Faculdade de Medicina de Ribeirão Preto* (CAAE 82637424.6.1001.5440) on November 12^th^, 2024. Informed consent will be required for all participants. The trial will be conducted in two pediatric intensive care units (ICUs): the coordinating center is an academic center, *Hospital das Clínicas* of *Faculdade de Medicina de Ribeirão Preto*, *Universidade de São Paulo*, which has an 8-bed pediatric ICU, and *Instituto de Oncologia Pediátrica* (IOP - GRAACC), which has a 19-bed pediatric ICU and is an oncologic center. The Brazilian Research Network in Pediatric Intensive Care (Brnet-PIC) supports the study.

Recruitment will begin in June 2025 and is expected to be completed by June 2026. The trial will follow the CONsolidated Standards Of Reporting Trials (CONSORT) guidelines, and the procedures will follow the ethical standards of the local committee on human experimentation and the Helsinki Declaration of 1975. The trial registration number is NCT06593288 (clinicaltrials.gov).

**Figure 1:**
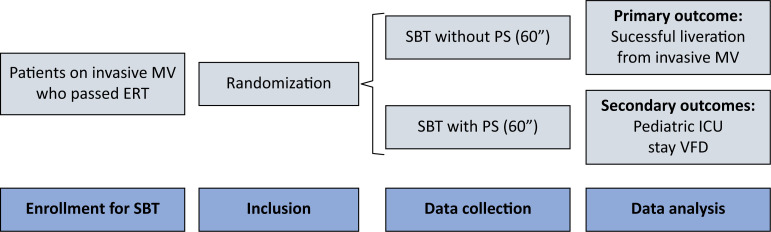
Schematic design of the trial.

Figure 1 shows the schematic design of the study.

MV - mechanical ventilation; ERT - extubation readiness test; SBT - spontaneous breathing trial; PS - pressure support; VFD - ventilation-free days.

### Eligibility criteria

#### Inclusion criteria

– Infants older than 1 month (> 36 weeks corrected gestational age) and children < 18 years old
– Admitted to the participating pediatric ICUs requiring invasive MV for more than 24 hours
– Clinically ready for weaning

#### Exclusion criteria

– Age < 1 month or > 18 years-old
– Chronic pulmonary conditions, congenital heart disease, or upper airway abnormalities
– Morbid obesity
– Palliative care patients
– Parental/guardian refusal

### Interventions

After passing the ERT, which is shown in table 1, patients will be randomized to either the PS or CPAP group. After consent, the local researcher will conduct randomization in REDCap electronic data capture tools hosted at the University of São Paulo. Both groups will undergo a 1-hour SBT. The PS group will receive PS adjusted according to the tracheal tube size, while the CPAP group will receive CPAP. The patient will be monitored during the SBT according to the worksheet in table 2.

**Table 1:** Extubation readiness test worksheet

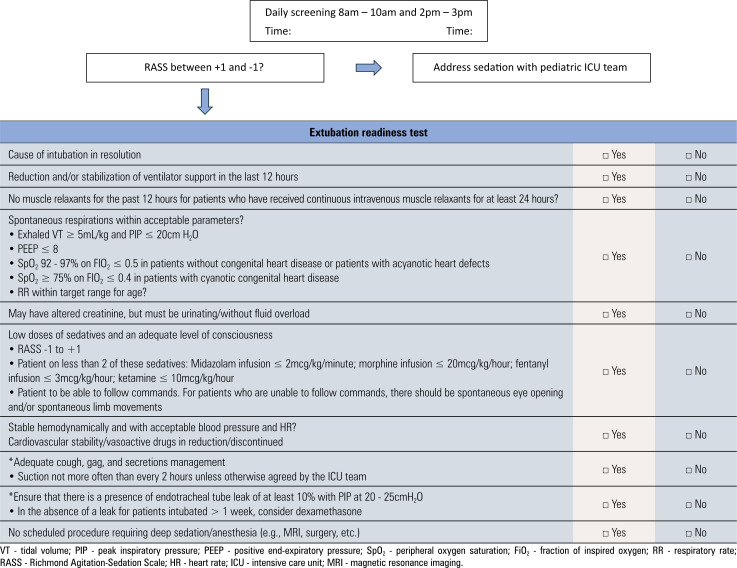

**Table 2:** Monitoring the patient during the spontaneous breathing test

Extubation readiness test		
Desaturation SpO_2_ < 92% in patients without congenital heart disease or patients with acyanotic heart defects SpO_2_ decreased by > 5% from baseline in patients with cyanotic congenital heart disease	□ Yes	□ No
RR increased above the normal range	□ Yes	□ No
Exhaled VT < 4mL/kg	□ Yes	□ No
Respiratory distress indicated by accessory muscle use, diaphoresis, and nasal flaring	□ Yes	□ No
Hemodynamic compromise HR ± 20% from baseline BP ± 20% from baseline	□ Yes	□ No
Patient went into apnea with ventilator backup ventilation	□ Yes	□ No
ETCO_2_ > 55mmHg or increase by 10mmHg or by 20% from baseline	□ Yes	□ No

If all the above checks in table 2 are NO at the end of a 1-hour spontaneous breathing test, inform the pediatric intensive care unit team of patient readiness for extubation, set a time for extubation, and decide on the post-extubation respiratory support level. If, for some reason, the patient is not extubated, justify:

SpO_2_ - peripheral oxygen saturation; RR - respiratory rate; VT - tidal volume; HR - heart rate; BP - blood pressure; ETCO_2_ - end-tidal carbon dioxide.

### SBT Groups

– **CPAP Group:** SBT for 1 hour with positive end-expiratory pressure (PEEP) and no PS. The PEEP and FiO_2_ will remain the same as during the ERT.
– **PS Group:** SBT for 1 hour with PEEP and PS, adjusted by tracheal tube size: PS = 6cmH_2_O for tubes > 5mm, 8cmH_2_O for tubes 4 - 5mm, and 10cmH_2_O for tubes ≤ 3.5mm.

### Primary outcome

Successful liberation from invasive MV is defined as no need for reintubation within 48 hours post-extubation.

### Secondary outcomes

– First SBT pass rate
– Need for post-extubation respiratory support (high-flow nasal cannula or noninvasive ventilation)
– Length of pediatric ICU stay

### Sample size and power calculation

Based on a previous study by Ferguson et al.,^(15)^ we estimate that 94 patients per group (188 total) will be needed to detect non-inferiority with 90% power and a 10% non-inferiority margin. Considering a 20% attrition rate, we plan to recruit 170 participants.

### Statistical methods

The primary analysis will be performed according to the intention-to-treat principle. Non-inferiority will be assessed using the Farrington-Manning test, with a 90% confidence interval to confirm the non-inferiority of CPAP compared to PS.

### Data collection and management

Data will be collected on patient demographics, clinical details, and ventilator settings, and stored in a secure online database (REDCap).

### Harms and adverse events

Adverse events will be documented, and serious adverse events will be reported to the ethics committee within 24 hours.

### Patient and public involvement

The research was developed to improve ventilation liberation in pediatric ICU settings, though patients were not involved in the design or recruitment process. Results will be disseminated through peer-reviewed publications.

## DISCUSSION

We present the design and rationale of the "Non-inferiority randomized controlled trial of SBT in children with and without pressure support", which aims to determine whether CPAP alone during an SBT is non-inferior to CPAP combined with PS in predicting successful extubation in mechanically ventilated children. The trial is designed as a multicenter, randomized, non-inferiority study, with the primary outcome being successful liberation from MV for at least 48 hours post-extubation. Secondary outcomes include the need for additional respiratory support post-extubation and the length of pediatric ICU stay. Protocol publication increases research transparency, facilitates future publication of results, avoids research duplication, and informs the community.

There is a scarcity of clinical trials in patients under MV, particularly on the best practices related to weaning and liberation from MV. This study will provide valuable data to guide clinical decision-making and influence future guidelines on weaning from MV in pediatric critical care.

A limitation of this trial is that the unblinded design may introduce bias. The 1-hour SBT duration is based on current practice, but this study may not address the optimal duration.

## Data Availability

The contents will be made available at the time of publication of the article.
